# Hospital Sunday

**Published:** 1874-08

**Authors:** 


### Editorial.

### Hospital Sunday.

It is, or should be the object of a Hospital to afford shelter and Medical or Surgical aid to those who from any circumstance are unable to procure it for themselves, or who cannot at their places of abode receive the care and attention necessary. Very few of our citizens we think, are aware of the work accomplished in our Hospitals, or of the calls made upon them for aid which are denied, simply for want of the necessary funds. To meet the pecuniary needs of the Buffalo General Hospital, an association of ladies, The Ladies Hospital Association, have in various ways endeavored to raise money. They have met with a slight measure of success, but there remains a large want to be supplied, and the history of this Institution is the history of every similar one in the city; daily some poor patient is turned away form its doors, simply because the means are not at hand for his support.

We have to suggest a plan which has been in successful operation in England, and also in one city at least in this country, by which the public could be made, in a measure acquainted with the wants of our Hospitals, and could be afforded an opportunity to contribute to their support. We refer to the establishment of what might be called "Hospital Sunday," on which day in all the Churches of the city, the Pastors could in a few remarks, present the claims of these institutions for support, and call upon their various congregations to contribute to that end.

Before this plan could be put into successful operation, some definite arrangements would be necessary, in reference to the time and mode of making the appeal, the distribution of the funds, etc, etc., but these matter could be arranged in a short time, and we hope soon to see the effort made in Buffalo. In Boston a few weeks since, over $13,000 was raised in the various churches and distributed among her hospitals. Cannot Buffalo respond in as loud a voice to an appeal which should reach every heart, and move every hand "pocketward." B.

